# Genome assembly of *Acinetobacter baumannii* strain 1432 carrying KL130 and eight prophage regions

**DOI:** 10.1128/mra.00454-25

**Published:** 2025-07-07

**Authors:** Anastasia S. Sukhova, Yury P. Skryabin, Peter V. Evseev, Angelika A. Sizova, Tatiana N. Mukhina, Anastasia Yu. Lebedeva, Anastasia V. Popova

**Affiliations:** 1 State Research Center for Applied Microbiology and Biotechnology, Obolensk, Russia; 2 Pirogov Russian National Research Medical University 64882, Moscow, Russia; Queens College Department of Biology, Queens, New York, USA

**Keywords:** *Acinetobacter baumannii*, genome assembly

## Abstract

We report the genome assembly of *Acinetobacter baumannii* strain 1432, a bacterial host for the previously described phage vB_AbaP_AS12, which was isolated from a burn patient in Nizhny Novgorod, Russia. The strain was found to carry capsule biosynthesis locus KL130. Eight prophage regions were bioinformatically predicted in the genome assembly.

## ANNOUNCEMENT

*Acinetobacter baumannii*, a non-fermentative gram-negative aerobic bacterium, is one of the most significant nosocomial pathogens (1) and has been assigned by the World Health Organization to the list of critically important bacterial pathogens to develop new strategies for antibiotic therapy (2). In this report, we announce the genome assembly of *A. baumannii* strain 1432 isolated from a burn patient in Nizhny Novgorod (Russia) in 2010. The strain is a bacterial host for the previously described phage vB_AbaP_AS12 (3). *A. baumannii* 1432 has been stored in the State Collection of Pathogenic Microorganisms and Cell Cultures of SRCAMB under accession number B-7134. Bacterial cells were grown at 37°C on Luria–Bertani agar (Difco Laboratories, USA) and Nutrient Medium No. 1 (Obolensk, Russia). DNA was extracted from a single colony grown overnight at 37°C using a DNeasy UltraClean Microbial Kit (Qiagen, Germany) according to the manufacturer’s protocol. Whole-genome sequencing (WGS) was performed on the MGISeq-2000 platform (MGI Tech Co., Ltd, China) using the MGIEasy Universal DNA Library Prep Set (MGI Tech Co., Ltd) with prefragmentation of DNA molecules by the BioRuptor System (Diagenode, USA), and the MGISEQ-2000 PE150 High-throughput Sequencing Kit (MGI Tech Co., Ltd). Up to 3,428,646 paired-end reads (1,028,593,800 bases) were generated by MGISeq-2000 with a total genome coverage depth of 259-fold. The genome assembly was performed using Unicycler v.0.5.0 software with default settings that included primary filtering and quality control (4). The final genome assembly consisted of 63 contigs and contained 4,330,643  bp with a GC content of 38.86% and an *N*_50_ value of 254,053 bp. The genome assembly was annotated with the NCBI Prokaryotic Genome Annotation Pipeline v.6.10 (5) and was found to contain a total of 4,220 genes, which are composed of 4,092 protein-coding sequences, 64 tRNA genes, and 56 pseudogenes. The CheckM2 v.1.1.0 analysis (6) estimated a genome completeness of 100%.

**Fig 1:**
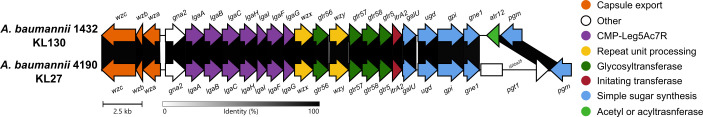
Comparison of KL130 and KL27. The arrows indicate genes in the transcription direction. The maps were created using Clinker (9). The scale bar and the sequence similarity percentage are indicated by the color intensity shown in the legend below. The color scheme is shown on the right. CMP, Cytidine monophosphate, Leg5Ac7R5,7-diamino-3,5,7,9-tetradeoxy-d-*glycero*-d-*galacto*-non-2-ulosonic (legionaminic) acid.

The identification of the capsule biosynthesis locus (K locus, KL) in the genome data were performed using Kaptive (7). It was previously shown that *A. baumannii* 1432 produces capsular polysaccharide (CPS) of K27 structure (8). After WGS analysis, the strain was found to carry KL130 (contig 10, positions 32,461–59,373). Both KL130 and KL27 (GenBank accession number KT266827) are very similar and share the same gene content, except for regions between genes *gne1* and *pgm* (Fig. 1). These differences most likely do not affect the final K27 CPS structure.

Prophage regions were identified using PHASTEST (10) with default settings and a sensitive Hidden Markov Model-based search HHblits (11) to detect genomic regions containing genes encoding the phage conserved proteins (major capsid protein and terminase large subunit). In total, eight distinct regions were identified (Table 1). BLAST and HHblits searches revealed the absence of genes encoding prophage-derived enzymes that degrade or modify CPSs in the *A. baumannii* 1432 genome assembly.

**TABLE 1:** General characterization of prophage regions in the *A. baumannii* 1432 genome assembly

No	Contig no	Region position	Region length (kb)	% GC	Completeness
1	4	1–26,528	26.5	38.6	Incomplete
2	7	200,817–235,450	34.6	38.5	Incomplete
3	12	14,305–22,940	8.6	31.4	Incomplete
4	14	1–107,113	107.1	38.9	Complete
5	16	1–26,594	26.6	39.6	Incomplete
6	26	1–23,259	23.3	40.3	Incomplete
7	29	1–10,872	10.9	41.7	Incomplete
8	31	1–9,968	10.0	41.5	Incomplete

## Data Availability

*Acinetobacter baumannii* 1432 genome sequence was deposited in the GenBank database under the BioProject accession PRJNA1256664 and BioSample accession SAMN48189432. The raw sequence reads have been deposited in the SRA under accession SRR33364854. Assembly is available with accession JBNJSI000000000.
